# THE EFFECT OF YOGA ON FALL PREVENTION OUTCOMES IN ADULTS 50 YEARS AND OLDER: A SYSTEMATIC REVIEW OF THE LITERATURE

**DOI:** 10.1093/geroni/igz038.1067

**Published:** 2019-11-08

**Authors:** Chinelo K Nsobundu, Margaret J Foster, Yan Hong

**Affiliations:** 1 Texas A&M University - School of Public Health, College Station, Texas, United States; 2 Texas A&M University, College Station, Texas, United States

## Abstract

Falls constitute a multitude of injuries irrespective of age. To combat these challenges, older adults are encouraged to engage in recreational activities. Yoga has been identified as an effective physical activity to promote mobility and balance for older adults. This study aims to systematically review the literature about yoga as a fall prevention intervention and synthesize the outcomes. Major databases (Ovid Medline & CINAHL) were searched for relevant articles. Studies were included if they met the criteria of 1) being a face to face yoga program, 2) aimed to recruit participants 50 years or older, and 3) reported at least one fall-related outcome (e.g., balance, mobility, fear of falling) as a result of the yoga program. 57 studies were identified: 32 from Ovid Medline and 25 from CINAHL. After removing the duplicates and applying a strict inclusion and exclusion criteria, 11 articles were included in the final analysis. A detailed synthesis of the results will be presented and quality assessment of included articles will be performed using the Modified Downs and Black checklist which appraises the methodological quality of both randomized and non-randomized studies. More research is needed to understand the impact of yoga in preventing falls among older adults at least 50 years of age. Additionally, research should establish a gold standard index that identifies which specific yoga programs ( based on type- individual vs. group; hatha, iyengar, kundalini, ashtanga, and etc.; frequency, and duration) have an enhanced effect on fall prevention.

